# Editorial: Multidisciplinary insights into distal tibiofibular joint injuries: an integrative analysis of surgical techniques, rehabilitation protocols, and podiatric interventions

**DOI:** 10.3389/fsurg.2025.1661487

**Published:** 2025-07-21

**Authors:** Roberto Tedeschi, Danilo Donati

**Affiliations:** ^1^ Independent Researcher, Bologna, Italy; ^2^ Clinical and Experimental Medicine PhD Program, University of Modena and Reggio Emilia, Modena, Italy; ^3^ Physical Medicine and Rehabilitation Unit, University Hospital Policlinico Sant'Orsola-Malpighi, Bologna, Italy

**Keywords:** tibiofibular joint, ankle instability, proximal fibula, regenerative orthopaedics, multidisciplinary care

## Abstract

This editorial explores recent advances in the understanding and treatment of tibiofibular joint disorders, integrating evidence from surgical, conservative, and regenerative approaches. Highlighting both distal and proximal dysfunctions, the contributions within this Research Topic reveal underrecognised biomechanical roles and therapeutic opportunities. From meta-analytical data on ankle instability to novel strategies in paediatric and osteoarthritic care, this collection advocates for anatomy-guided, patient-specific interventions. A structured summary of clinical outcomes and evidence levels is provided to guide future interdisciplinary efforts in orthopaedic and podiatric practice.

**Editorial on the Research Topic**
Multidisciplinary insights into distal tibiofibular joint injuries: an integrative analysis of surgical techniques, rehabilitation protocols, and podiatric interventions

The tibiofibular complex—both proximal and distal—is a biomechanical continuum essential for load transmission, ankle stability, and coordinated lower limb function. Yet, its pathologies remain underappreciated in clinical practice, often overshadowed by more prominent articular disorders. This Research Topic brings forward a multidisciplinary perspective on these clinically impactful yet frequently underexplored regions, offering an integrated view across anatomy, pathology, surgical innovation, conservative strategies, and regenerative approaches.

A recurring clinical challenge concerns the management of chronic lateral ankle instability, where the optimal extent of surgical reinforcement remains debated. Some procedures routinely incorporate augmentation techniques, such as inferior extensor retinaculum (IER) reinforcement, despite a lack of conclusive evidence on added functional benefit (Liu et al., 1). The evidence emerging from recent comparative analyses suggests that, in selected cases, such reinforcement may not substantially improve outcomes compared to standard ligament repair. These findings invite a rethinking of traditional surgical dogma, emphasising the need for lesion-specific approaches guided by anatomical severity rather than procedural habit.

Parallel to this, the broader biomechanical context of the fibula—beyond its role in ankle stabilization—has gained attention for its impact on knee mechanics. The proximal tibiofibular joint (PTFJ), a synovial articulation situated just below the lateral tibial plateau, has long been neglected in musculoskeletal models. However, its role in axial load modulation, fibular motion, and peroneal nerve dynamics has now been better delineated (Tianjun et al.). Recent anatomical and clinical syntheses have highlighted how dysfunction at this level, whether due to instability, degeneration, or variant morphology, may contribute to lateral knee pain, restricted mobility, and referred neuropathic symptoms (2). Emerging surgical options such as proximal fibular osteotomy (PFO), applied in cases of medial knee osteoarthritis, propose a novel load-redistribution strategy rooted in the concept of “uneven settling” (Tianjun et al., 3). These interventions, once marginal, are now supported by early clinical outcomes and offer new therapeutic targets within a complex anatomical and functional framework.

In the domain of trauma, the management of complex hindfoot fractures—particularly Sanders type II–III calcaneal fractures—exemplifies the shift toward minimally invasive fixation strategies. Techniques such as percutaneous cross-bar external fixation, when compared to traditional open approaches, show promise in reducing operative time, postoperative pain, and soft-tissue complications while maintaining articular alignment and functional outcomes (Wang et al., 4). Such approaches reflect a broader trend in orthopaedic surgery: achieving stability through biomechanically sound constructs without the morbidity of extensive dissection.

This same principle—preservation of biological and vascular environments—underpins advances in the management of recalcitrant tibial non-unions. In cases where multiple surgical attempts have failed, integrating the “diamond concept” has proven effective: combining mechanical stability with osteogenic grafting, growth factor stimulation, and periosteal coverage to create a chamber conducive to bone healing (Wang et al., 5). The enrichment of autologous bone with platelet-rich plasma (PRP), along with the strategic containment of the graft in a vascularised niche, represents a compelling example of regenerative orthopaedics in practice.

From a developmental perspective, the syndesmotic region also presents unique challenges in paediatric populations. Conditions such as fibular hemimelia, often approached through early reconstructive surgery, may benefit from conservative protocols in select cases (Ma et al.). Evidence suggests that neonatal splinting can, in specific phenotypes, support spontaneous joint stabilization and permit delayed limb lengthening without the need for early surgical intervention. These findings prompt a reevaluation of rigid treatment sequences in congenital deformity, highlighting the potential of growth-guided conservative approaches when applied with precision and clinical foresight.

To provide a concise synthesis of the clinical impact and methodological quality of the contributions, Table 1 summarises the primary outcomes and the relative strength of evidence across studies included in this Research Topic. Across diverse clinical contexts—instability, trauma, osteoarthritis, deformity, or non-union—the articles underscore the importance of tailoring interventions to the anatomical, biomechanical, and biological specificities of each case. Rather than adhering to rigid procedural hierarchies, the emphasis shifts toward integrated reasoning grounded in structure-function relationships.

**Table 1:** Clinical outcomes and strength of evidence.

Main outcome(s)	Strength of evidence
No added benefit from IER augmentation; functionally equivalent outcomes	High (Level 1)
PTFJ dysfunction linked to knee pain; PFO as therapeutic option	Moderate (Expert consensus, Level 5)
Lower pain and complication rate with similar alignment outcomes	Moderate (Level 3)
Successful union after 6 failed surgeries using diamond concept	Low (Level 4)
Stability achieved with splinting; delayed surgery possible	Low (Level 4)

Moreover, a consistent methodological message emerges: multimodal assessment is key. Whether through advanced imaging for PTFJ pathology (Tianjun et al.), meta-analytical aggregation for surgical decisions (Liu et al., 6), or functional metrics in outcome evaluation (Wang et al.), these studies promote a standard of care that is both data-informed and biologically coherent. The fibula, long seen as a passive stabiliser, reclaims its role as an active participant in lower limb dynamics, deserving of more nuanced clinical and research attention.

This collection of articles does not aim to exhaust the topic but to set a foundation for future research. There is a growing need for multicentric studies, biomechanical simulations, and high-level trials that explore not only joint repair but also functional restoration and long-term durability. The integration of regenerative medicine, load-sensitive fixation technologies, and personalised rehabilitation protocols may define the next generation of care for patients with tibiofibular disorders.

Ultimately, the clinical complexity of the tibiofibular joints calls for cross-disciplinary collaboration: orthopaedic surgeons, physiotherapists, podiatrists, anatomists, and pain specialists must work in concert to navigate the subtleties of diagnosis and treatment. This Research Topic provides a platform for such integration, reinforcing the concept that high-quality care emerges not from isolated innovation, but from shared insight across domains.
